# Modeling neurodegeneration in the retina and strategies for developing pan-neurodegenerative therapies

**DOI:** 10.1186/s13024-025-00858-5

**Published:** 2025-10-14

**Authors:** Emily L. Ward, Larry Benowitz, Thomas M. Brunner, Guojun Bu, Michel Cayouette, Valeria Canto‐Soler, Sandro Dá Mesquita, Adriana Di Polo, Aaron DiAntonio, Xin Duan, Jeffrey L. Goldberg, Zhigang He, Yang Hu, Shane A. Liddelow, Anna La Torre, Milica Margeta, Francisco Quintana, Karthik Shekhar, Beth Stevens, Sally Temple, Humsa Venkatesh, Derek Welsbie, John G. Flanagan

**Affiliations:** 1 Herbert Wertheim School of Optometry & Vision Science, University of California Berkeley, Berkeley, CA USA; 2 Vision Science Graduate Group. Herbert Wertheim School of Optometry & Vision Science, University of California Berkeley, Berkeley, CA USA; 3 Department of Neurosurgery and F.M. Kirby Neurobiology Center, Boston Children’s Hospital, Harvard Medical School, Boston, MA USA; 4 Department of Ophthalmology, University of Pittsburgh, Pittsburgh, PA USA; 5 Glaucoma Research Foundation, San Francisco, CA USA; 6 Division of Life Science, Hong Kong University of Science and Technology, Hong Kong, China; 7 Molecular Biology Program, Université de Montréal, Montreal, Canada; 8 Cellular Neurobiology Research Unit, Institut de Recherches Cliniques de Montreal (IRCM), Montreal, Canada; 9 Department of Medicine, Université de Montréal, Montreal, Canada; 10 Department of Anatomy and Cell Biology, Division of Experimental Medicine, McGill University, Montreal, Canada; 11 CellSight Ocular Stem Cell and Regeneration Research Program, Department of Ophthalmology, University of Colorado School of Medicine, Sue Anschutz-Rodgers Eye Center, Aurora, CO USA; 12 Department of Neuroscience, Mayo Clinic, Jacksonville, FL USA; 13 Department of Neuroscience, University of Montreal, Montreal, QC Canada; 14 Needleman Center for Neurometabolism and Axonal Therapeutics, St. Louis, MO USA; 15 Department of Developmental Biology, Washington University School of Medicine, St. Louis, MO USA; 16 Department of Ophthalmology, University of California San Francisco, San Francisco, CA USA; 17 Spencer Center for Vision Research, Byers Eye Institute, Stanford University, Palo Alto, CA USA; 18 F.M. Kirby Neurobiology Center, Boston Children’s Hospital, Boston, MA USA; 19 Neuroscience Institute, NYU Grossman School of Medicine, New York, NY USA; 20 Department of Cell Biology and Human Anatomy, University of California Davis, Davis, CA USA; 21 Department of Ophthalmology, Harvard Medical School, Mass Eye and Ear, Boston, MA USA; 22 Department of Neurology, Brigham and Women’s Hospital, Harvard Medical School, Boston, MA USA; 23 Department of Chemical and Biomolecular Engineering, Helen Wills Neuroscience Institute, University of California Berkeley, Berkeley, CA USA; 24 Department of Neurology, F.M. Kirby Neurobiology Center, Howard Hughes Medical Institute, Boston Children’s Hospital, Harvard Medical School, Boston, MA USA; 25 Neural Stem Cell Institute, Albany, NY USA; 26 Shiley Eye Institute and Viterbi Family Department of Ophthalmology, University of California, San Diego, CA USA

**Keywords:** Glaucoma, Neurodegeneration, Alzheimer’s Disease, Parkinson’s Disease, Neuroprotection, Neuroinflammation, Glia, Retinal ganglion cells, Retinal pathologies, Amyotrophic lateral sclerosis

## Abstract

**Background:**

Glaucoma Research Foundation's third Catalyst for a Cure team (CFC3) was established in 2019 to uncover new therapies for glaucoma, a leading cause of blindness. In the 2021 meeting “Solving Neurodegeneration,” (detailed in Mol Neurodegeneration 17(1), 2022) the team examined the failures of investigational monotherapies, issues with translatability, and other significant challenges faced when working with neurodegenerative disease models. They emphasized the need for novel, humanized models and proposed identifying commonalities across neurodegenerative diseases to support the creation of pan-neurodegenerative disease therapies.

Since then, the fourth Catalyst for a Cure team (CFC4) was formed to explore commonalities between glaucoma and other neurodegenerative diseases. This review summarizes outcomes from the 2023 “Solving Neurodegeneration 2” meeting, a forum for CFC3 and CFC4 to share updates, problem solve, plan future research collaborations, and identify areas of unmet need or opportunity in glaucoma and the broader field of neurodegenerative disease research.

**Main body:**

We summarize the recent progress in the field of neurodegenerative disease research and present the newest challenges and opportunities moving forward. While translatability and disease complexity continue to pose major challenges, important progress has been made in identifying neuroprotective targets and understanding neuron-glia-vascular cell interactions. New challenges involve improving our understanding of the disease microenvironment and timeline, identifying the optimal approach(es) to neuronal replacement, and finding the best drug combinations and synergies for neuroprotection. We propose solutions to common research questions, provide prescriptive recommendations for future studies, and detail methodologies, strategies, and approaches for addressing major challenges at the forefront of neurodegenerative disease research.

**Conclusions:**

This review is intended to serve as a research framework, offering recommendations and approaches to validating neuroprotective targets, investigating rare cell types, performing cell-specific functional characterizations, leveraging novel adaptations of scRNAseq, and performing single-cell sorting and sequencing across neurodegenerative diseases and disease models. We focus on modeling neurodegeneration using glaucoma and other neurodegenerative pathologies to investigate the temporal and spatial dynamics of neurodegenerative disease pathogenesis, suggesting researchers aim to identify pan-neurodegenerative drug targets and drug combinations leverageable across neurodegenerative diseases.

**Graphical Abstract:**

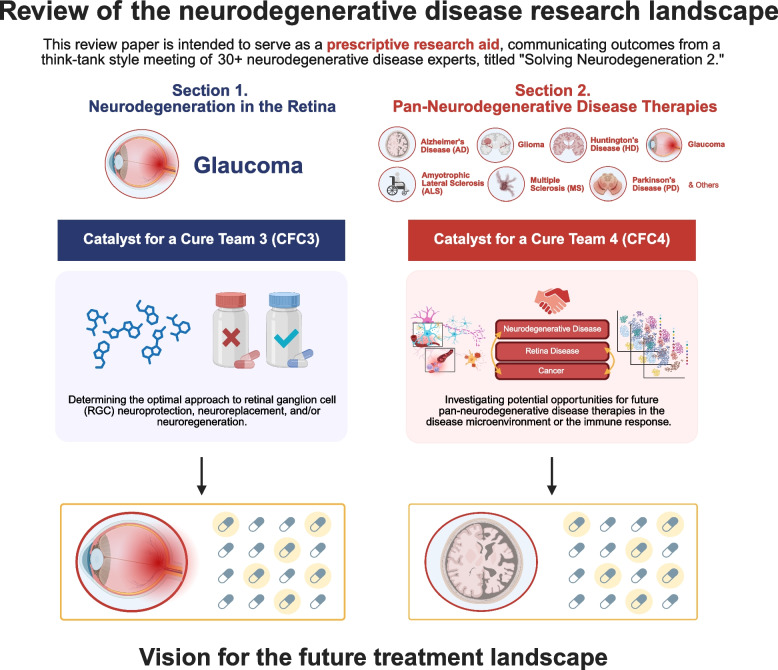

## Background

This review aims to promote progress in neurodegenerative disease research by reviewing the major advancements, challenges, and future directions in the present research landscape. Throughout this review, we discuss the ongoing and future contributions by the Glaucoma Research Foundation’s latest Catalyst for a Cure (CFC) research programs: CFC Team 3 and CFC Team 4. Two common areas of focus include dissecting the neurodegenerative disease microenvironment and elucidating the role of the immune response. We consider how these factors relate to neurodegenerative disease pathogenesis and progression, including across space (e.g., anatomical location) and time (e.g., stage of disease), and how this information may be leveraged into potential future therapeutic strategies for stopping, preventing, or reversing neurodegenerative diseases. As an example, one major initiative aims to determine and interpret the commonalities and differences in the disease microenvironment across different neurodegenerative diseases, on the basis that future pan-neurodegenerative disease therapies could potentially be developed against common underlying features or disease mechanisms and prescribable across different diseases. Investigators also hope to add knowledge and future treatment options by investigating the microenvironment and immunity across other variables, including different disease subtypes and individual patients' differences. In a more distant future, these lines of inquiry could potentially enable the development of targeted therapies and personalized medicine for neurodegenerative diseases. Importantly, this general framework focused on the microenvironment and immunity has been historically applied in cancer research, successfully contributing to the advent of many treatment options and improving cancer survival rates [1].

### Critical need for treatments

Neurodegenerative diseases pose an increasingly critical threat to global public health. Glaucoma, Alzheimer's Disease (AD), Parkinson's Disease (PD), amyotrophic lateral sclerosis (ALS), multiple sclerosis (MS), and Huntington's Disease (HD) are among the most common types of neurodegeneration, which is characterized by the progressive and irreversible death of neurons. Neurodegenerative diseases are associated with high incidence, poor prognosis, and profoundly negative impact on quality of life [2, 3]. As life expectancies rise worldwide and the global population continues to age rapidly, healthcare systems are already under pressure to adapt to and manage a growing population of patients with age-related neurodegenerative disease and other chronic conditions [4, 5].

Individuals aged 65 and older are at higher risk of AD and other forms of neurodegeneration compared with their younger counterparts [4]. Neurodegenerative disease diagnoses will more than triple by 2050 [4, 5], and by 2060, the number of Americans aged 65 and older will more than double [6]. While recent decades have seen improved survival rates among patients with cancer, cardiovascular disease, and other leading causes of death, mortality is rising among patients with AD, PD, and other forms of neurodegeneration [2, 4, 7]. And while treatments exist for cancer and cardiovascular disease, to date, no therapies are approved to halt or reverse neurodegeneration. The urgency to address the neurodegenerative disease epidemic is furthered by the growing evidence linking its molecular pathophysiology to common lifestyle factors, such as poor diet and low physical activity [8]. More than one-third of neurodegenerative diseases cases in the U.S. are associated with obesity, physical inactivity, and/or other modifiable risk factors [4].

**Fig. 1:**
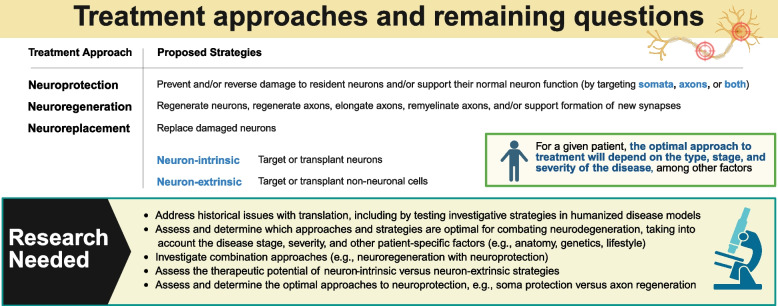
Treatment approaches and remaining questions

The heterogeneity, pleiotropy, and overall complexity of neurodegenerative diseases have hindered the development of effective therapies to date. Individual differences in disease pathogenesis and presentation further complicate the development of effective therapies, and individual responses to quality-of-life interventions are variable and often insufficient. While many potential drug targets have been identified, even promising investigational monotherapies have failed to translate from animal models into humans [9–11]. Rather, it has become increasingly evident that the optimal approach to treating neurodegeneration will depend on the stage or severity of the disease and other underlying, patient-specific factors, such as their anatomy or genetics. In many cases, a combination of treatment regimens may be necessary to halt the disease progression. Among other burdens associated with neurodegenerative disease, these significant unmet needs underscore the immediate need to develop innovative approaches to disease prevention and intervention. In general, the proposed strategies for treating neurodegeneration are: neuroreplacement, neuroregeneration, and neuroprotection (Fig. 1).

### Catalyst for a Cure

The Glaucoma Research Foundation launched its flagship research initiative, Catalyst for a Cure, in 2002 to accelerate progress toward vision restoration and a cure for glaucoma, a group of optic neuropathies. Catalyst for a Cure was among the first programs to unite leading glaucoma specialists and neuroscientists in a collaborative model designed to drive innovation through shared expertise. The first and second CFC teams yielded major advances in glaucoma research, setting the stage for continued progress [12]. The subsequent teams have expanded the program’s scope, integrating insights from cancer biology and other neurodegenerative diseases.

As of 2025, the active Catalyst for a Cure research campaigns are CFC Team 3 and Team 4. Catalyst for a Cure Team 3 was established in 2019 to identify new therapeutic strategies for glaucoma. Since then, the fourth Catalyst for a Cure team was formed to explore commonalities between glaucoma and other neurodegenerative diseases. Members of each CFC team work closely throughout the year and convene annually at “Solving Neurodegeneration,” a private roundtable meeting where investigators present research updates, exchange ideas, and receive feedback from peers and a scientific advisory board.

### Solving Neurodegeneration

In April, 2021, the Glaucoma Research Foundation, BrightFocus Foundation, and the Melza M. and Frank Theodore Barr Foundation convened key opinion leaders and expert investigators for the first iteration of the “think-tank” style meeting, Solving Neurodegeneration. The attendees concluded that mechanisms common across neurodegenerative diseases must be identified and investigated for their potential to aid in the creation of neurodegenerative treatments and treatment combinations. The experts also recommended using humanized disease models and leveraging natural developmental processes for cell replacement and regeneration [11].

This review article was developed following the August, 2023 meeting “Solving Neurodegeneration 2,” summarizing the latest advancements, challenges, and new directions facing facing the field of neurodegenerative disease research, and presenting the latest perspectives, recommendations, and contributions from the Catalyst for a Cure team researchers, the meeting’s keynote speakers, and the CFC scientific advisory board. The initial focus is on retinal ganglion cell (RGC) neurodegeneration, with implications for therapeutic strategies including neuroprotection, neuroregeneration, and cellular replacement for glaucoma. As part of this coverage, we discuss recent work of Catalyst for a Cure Team 3, who are searching for glaucoma treatment strategies. We then extend this review coverage to other neurodegenerative diseases, discussing shared disease mechanisms and exploring potential strategies for future pan-disease therapies, and including coverage of Catalyst for a Cure Team 4.

## Neurodegeneration in the retina

Glaucoma is a collective term for a group of optic neuropathies. In glaucoma, there is neurodegeneration of retinal ganglion cells (RGCs), the class of neurons whose axons form the optic nerves and carry signals from the retina to the brain. Glaucoma is one of the most common neurodegenerative diseases in the United States and worldwide [13, 14]. Approximately 4.22 million people in America had glaucoma in 2022 (1.62% of adults; 2.56% of people 40 years or older) [15]. The estimated global prevalence is 80 million people, and that number is expected to rise over 111 million by 2040 [14].

It is a leading form of irreversible blindness and a major threat to public health [16, 17]. The most common types of glaucoma often go clinically undetected until the disease has caused irreversible damage to the eye and progressive vision loss has begun. Around 50% of glaucoma cases are clinically undetected and the true prevalence is likely higher than estimated [18–21].

The article from “Solving Neurodegeneration” 2021 emphasized the need to develop novel research models for glaucoma, investigate neuroprotection and neuroreplacement strategies, and identify commonalities between glaucoma and other neurodegenerative diseases [11]. The following section provides a progress update in these areas and reviews the latest achievements, challenges, and future directions for the field of glaucoma research. We also present insights that may be relevant to the study of other neurodegenerative diseases. For example, studies in the retina can provide insights into central nervous system (CNS) development, maintenance, degeneration, and repair. Similarly, studying neurodegeneration in the retina may reveal insights relevant to the development of pan-neurodegenerative disease therapies and the larger quest to solve neurodegeneration [22].

### Retinal ganglion cell (RGC) replacement

A major aim in glaucoma research is developing strategies to restore vision by replacing lost RGCs. This section discusses recent advancements in testing cell transplantation and cell reprogramming as strategies for RGC replacement.

#### Transplantation as an RGC-replacement strategy

Researchers are investigating a novel model for RGC replacement that will involve transplanting donor RGCs from retinal organoids into non-human primates [23], building on their previous success using this model in rodents [23]. A current challenge in improving the model is increasing the number of transplanted cells. The location and precision of the transplantation are also difficult to control. Thus, future studies will aim to improve the ability of the transplanted cells to cross the inner limiting membrane [23].

Another significant challenge will be ensuring that the transplanted RGCs extend their axons to the optic nerve head (ONH), into the optic nerve, and through the lamina cribrosa. Individually, the ONH, the pre-laminar optic nerve, the lamina cribrosa, and the post-laminar optic nerve are unique microenvironments of the CNS, each of which may present specific challenges to transplantation as a neuroreplacement strategy. For example, the cellular composition, structure, and biomechanical properties of the ONH differentiate it from the other areas of the retina or the optic nerve. The lamina cribrosa is also unique in its cellular composition, anatomy, biomechanics, and vascular supply. Researchers therefore must consider the unique challenges in each of these anatomical locations, including but not limited to potential challenges related to endogenous tissue structure density/complexity, the presence of axonal support cells (such as astrocytes and/or oligodendrocytes), biomechanical forces (such as stretch, shear, or strain), ensuring adequate blood supply, the potential for adverse vascular events, and immune surveillance (including innate CNS immunity versus peripheral invading immunity). In future, RGC replacement and restoring visual function will require our ability to induce or guide cellular growth in each of these areas with consistency and precision. Therefore, it will be important to consider the unique qualities and challenges of these sites and other microenvironments along the visual pathway.

After exiting the eye, axons of transplanted RGCs should travel through the optic nerve, terminating in synapses within specific areas of the lateral geniculate nucleus and enabling visual function. This challenge will likely be addressed using genetic tools to increase our understanding of how RGCs wire from retina to brain during normal development [23]. Eye-to-brain connectivity and other challenges to RGC repopulation are thoroughly detailed in Soucy et al. [24].

Future research in this area will include the use of spatial transcriptomics to determine the sensitivity or resiliency of distinct subtypes of transplanted RGCs. Additionally, Luo & Chang provides a helpful review of the advantages and disadvantages of differential protocols for stem-cell-derived RGC replacement [25].

#### Cell reprogramming as an RGC-replacement strategy

Cell reprogramming is another proposed strategy for replacing RGCs lost to glaucoma. Cell reprogramming refers to controlled neurogenesis, the progression of neural stem cells (NSCs) and neural progenitor cells (NPCs) into functionally integrated mature neurons. This strategy is centered on the idea that donor stem/progenitor cells could be transplanted into the retina and differentiated into new RGCs [26, 27]. To this end, one recent area of focus has been investigating retinal organoids as potential sources for donor stem/progenitor cells. Researchers have also proposed that it may be possible to reprogram resident retina cells into new RGCs, altogether avoiding the need for transplantation.

##### Studying CNS development enables cell reprogramming

Studying CNS development and deepening our ability to control neurogenesis may one day enable the therapeutic use of cell reprogramming as a viable strategy to replace cells lost to neurodegenerative disease. During neurogenesis, the timing and spatial arrangement of stem/progenitor cell differentiation are intrinsically related to the differentiated cell fate. Researchers have identified certain gene regulatory networks that control the timing and spatial arrangement of cell fates during development [28–30]. For example, distinct networks regulated by sonic hedgehog morphogens and TGFβ respectively dictate the spatial and temporal patterning of neural stem cells in the spinal cord [29, 30]. Studying the spatial and temporal patterning of CNS development is crucial, as identifying distinct gene regulatory networks that control these aspects enhances the potential to therapeutically manipulate the fates of newly generated cells in the CNS, including their cell type identity, wiring, and location, potentially enabling the replacement of cells lost to neurodegenerative disease [23, 31].

##### Studying development in the retina

The retina can serve as a useful tissue for studying CNS development and investigating cell reprogramming as a potential therapeutic strategy. For example, our understanding of the development process in the vertebrate CNS has increased dramatically across the last two decades based on studies performed in the mouse retina [32, 33]. Studies investigating the mouse retina provided the first evidence in the vertebrate CNS of molecular mechanisms orchestrating spatial and temporal control over neurogenesis and cell fate acquisition [32–35]. These studies demonstrate that researchers can extrapolate information about neurogenesis and cell fate by modeling and investigating their questions in the retina. Given that cell reprogramming could be a viable approach to replacing cells lost to neurodegeneration, and given that cell reprogramming can be enabled by uncovering and controlling molecular factors which control neurogenesis/cell fate acquisition (Table 1), it follows that the retina can be useful tool for investigating questions related to cell reprogramming and cell replacement strategies for neurodegenerative disease more generally.

**Table 1:** Temporal identity factors for controlled neurogenesis

Temporal Identity Factors		Select Literature
***Pou3f1*** **, ** ***Atoh7***	Drive RGC axon projections contralaterally (*Pouf31*) or contralaterally and ipsilaterally (*Atoh7*)	[36]
***Numb1***	Regulates Tau	[37]
***Myt1L***	Represses non-neuronal lineages, e.g. used in fibroblasts	[38] [39]
***Brn2***	Pro-neurogenic factor, e.g. used in fibroblasts	[38] [39]
***Ascl1***	Reprogramming factor, e.g. used in fibroblasts	[38–40]
***Ikzf1, Ikzf4*** **(Ikaros family)**	Reprogramming terminally differentiated cells, e.g., Müller glia	[33, 34, 36, 41]
***Pou2f1, Pou2f2***	Reprogramming terminally differentiated cells	[35]
***Islet1, Pou2f1/2, Pou4f2 (Brn3b), Atoh1, Okarps, Casz1, bhLH, Sox2,*** ***Tgfβ*, *Nrl/Nr2e3***	Other useful temporal identity genes	[28, 29, 32, 35, 40, 42, 43]

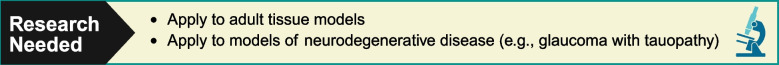

The retina can also be a useful model due its well-understood organization, circuitry, and cell composition, with available genomic profiles for its seven major cell classes [32–35, 43–45]. The retina is also relatively visualizable in vivo and ex vivo compared with other parts of the CNS. In vivo, depending on the type of information desired, many features of the retina can be imaged directly using tools such as optical coherence tomography (OCT) and retinal angiography. Ex vivo, while complex gray matter structures in the brain require multidimensional analyses, the retina can be flat-mounted and treated like a two-dimensional surface, including through the use of visualizable research tools (e.g., immunohistochemistry or optogenetics). Individual parts of the retina can be specifically assessed using dissection methods that isolate anatomical regions. A cross-sectioned retina can be used to visualize cells in any of the six layers, and staining can be localized to specific layers of the retina by targeting certain cell types [46].

**Fig. 2:**
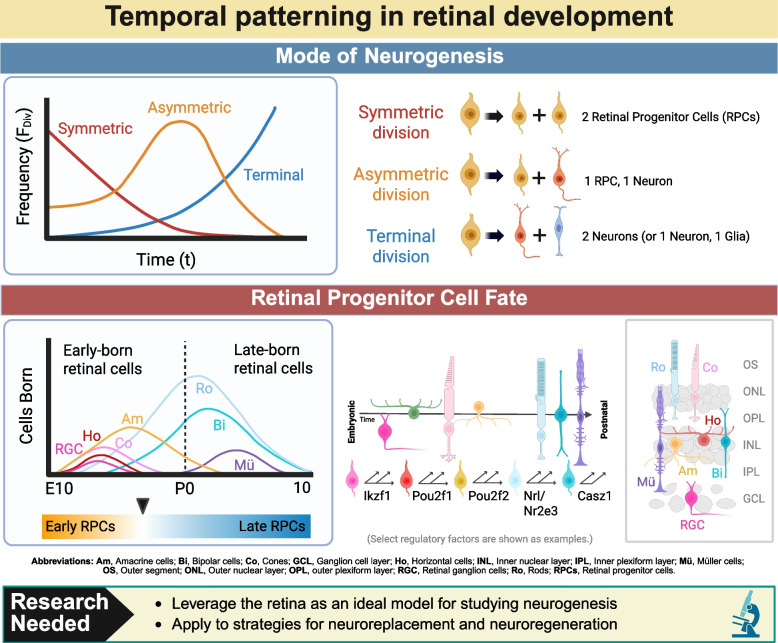
Temporal patterning in retinal development

In addition to its structural features, the retina can also be a convenient model for studying development because its neurons and Müller glia derive from a single population of multipotent progenitor cells, which differentiate into retinal cells according to a conserved sequence (Fig. 2) [47]. In the brain and spinal cord, combinatorial codes of regulatory gene networks are associated with greater progenitor cell diversity and increased complexity in temporal and spatial gene expression patterning [48–50]. While the evidence suggests that spatial patterning during retinal development is generally controlled by gradients of morphogens, temporal patterning appears more complex [30].

Temporal patterning controls the dominant mode of retinal progenitor cell division and the identities of the resulting retinal cell types (Fig. 2). Following a conserved sequence, retinal ganglion cells, horizontal cells, cones and GABAergic amacrine cells are generated at early (embryonic) stages of development, while Glycinergic amacrine cells, rod photoreceptors, bipolar cells and Müller glia are generated at late (postnatal) stages of retinal development [47]. In coordination with these changes, retinal progenitor cells also shift their modes of division from proliferative to neurogenic division. Asymmetric division dominates midway through the process of neurogenesis, and terminal division dominates at later time points [51, 52]. To better understand temporal patterning in the CNS, genomic studies are being used to identify the gene regulatory networks acting during particular windows of retinal development [23, 29, 36].

##### Temporal patterning factors for cell reprogramming

Studies have characterized the expression patterns and gene regulatory networks at play during different time-points of retinal development, producing numerous sc/snRNAseq and snATACseq datasets from mouse and human samples [23, 29, 36, 51, 53–56]. Several transcription factors have now been implicated in either the regulation of competence progression or the regulation of genetic programs that establish specific lineages, and these factors can be leveraged to develop retinal progenitors into specific retinal neurons [32–35, 43, 44]. Critical pathways that temporally regulate retinal development include retinoic acid, FGF, Foxp1, and Notch [57–59] Other temporal regulatory networks include Ikaros/Hunchback and Castor (identified in *Drosophila*) [60] and heterochronic miRNAs (identified in *C. elegans*) [61].

##### Progress using cell reprogramming for RGC replacement

Through preclinical studies, researchers have demonstrated the ability to regenerate CNS cells by leveraging key development pathways responsible for dictating stem cell fates [27, 38, 40]. Their work suggests that cell reprogramming may be a viable therapeutic strategy for replacing cells in the degenerating CNS. 

In healthy and damaged/diseased models of the retina, researchers have successfully transformed resident cells into new retinal neurons [38, 40], suggesting a similar therapeutic approach might enable RGC replacement while avoiding the need for cell transplantation. Recent work in this area has demonstrated that our ability to control retinal development is becoming increasingly specific; In one model of induced RGC differentiation, controlling the expression of *Pou3f1* expression can drive the specific development of contralateral RGCs [62]. Further studies will test whether *Pouf31* promotes full cellular differentiation and projection of axons into the brain [36]. Additionally, neuron-like cells have been generated from resident prenatal fibroblasts, [38] and Müller glia have been transformed into RGC-like cells (important factors include *Ascl1, Islet1, Pou4f2 (Brn3b), Atoh1, Myt1L, Brn2*) [40]. These pathways require future investigation in adult tissue models and various models of glaucoma, including those with tauopathy. In mouse models of tauopathy, postnatal RGCs can be reprogrammed using the developmental *Numb1* gene to decrease tau production and provide structural and functional neuroprotection [36, 37].

In summary, these recent studies suggest that controlled neurogenesis may be a viable strategy for RGC replacement [23, 26, 27, 31]. The growing availability of genetic datasets on retinal development has enhanced our understanding of temporal patterning during neurogenesis, and the field is quickly advancing its ability to develop new retinal neurons using known regulatory factors [32–35, 43, 44]. However, less clear are the combinatorial mechanisms underlying temporal patterning and its control over retinal cell fate. Currently, there is conflicting evidence regarding the extent to which certain factors may regulate both progenitor cell competence and progenitor cell division. The retina remains a strong model for studying these important aspects of CNS development. In the future, it may be possible to extrapolate this proof-of-concept to other regions of the CNS, including by drawing upon existing literature related to gene regulatory networks for controlled neurogenesis in other parts of the CNS.

### RGC neuroprotection and axon regeneration

In the previous section, we reviewed the recent glaucoma literature on investigative approaches to replacing and regenerating neurons lost to neurodegeneration. However, neuroregenerative treatments alone will be insufficient for treating neurodegenerative disease. Treatments are also needed to slow, stop, or reverse the underlying neurodegeneration (Fig. 1). Glaucoma and other neurodegenerative diseases are most commonly diagnosed after irreversible tissue damage has already begun. Therefore, there is an urgent need to develop neuroprotective treatment strategies aimed at protecting and supporting the remaining resident neurons during disease.

The proposed strategies for neuroprotection are based on preventing or reversing the damage to resident neurons and/or support their normal neuron function. Distinct investigative approaches to neuroprotection can be associated with different benefits, challenges, and future research directions. In this section, we summarize the recent progress made in studies investigating RGC neuroprotection and axon regeneration, including targeted approaches and transplant-based approaches. Conventional outcome measures used to measure RGC neuroprotection include measures based on RGC viability, axon integrity, or function.

#### Targeted approaches to RGC neuroprotection

##### Targeting the soma, the axon, and/or the dendrites

Investigative strategies for neuroprotection can be informed by studying the temporal dynamics of neuron survival and injury in the CNS. For example, some proposed strategies for neuroprotection utilize pathways intrinsically related to neuronal development, growth, or repair, such as the complement system (e.g., C1q, C3), Sonic Hedgehog, or Notch [63–66]. Other opportunities for neuroprotection derive from investigating pathways involved in physiological synapse pruning, including complement, or clearing of neuronal debris, such as LC3 [67]. Another approach to achieving neuroprotection focuses on pathways directly involved in axon destruction (e.g., DLK/JUN, SARM1) [31, 68–70] or growth/regeneration.

Early features of neurodegeneration include challenges to axonal physiology, axon transport, axon ultrastructure [71], and axon degeneration (i.e., axonopathy). Axons are metabolically demanding due to the substantial energy required for neurotransmission and the subcellular trafficking of molecules and organelles. Axon survival is therefore closely linked to energy metabolism. Additionally, relative to somata, long-range axons are vulnerable to injury, neurotoxin exposure, or issues with protein turnover (e.g., lysosomal dysfunction and plaque formation) [72]. Therefore, it remains to be determined whether the optimal approach to achieving neuroprotection should be aimed at protecting the RGC dendrites, the cell bodies (the somata), the axons, or a combination of these cellular components. It also remains to be determined whether and how the optimal approach may vary across factors, such as the type of neurodegenerative disease, the stage of disease, or individual patient differences, as emphasized in Fig. 1.

For example, the Bcl2-associated X protein (Bax) specifically contributes to the degeneration of RGC dendrites in glaucoma. Genetic deletion of Bax (*Bax-/-*) in models of glaucoma prolongs the survival of RGC somata—although axonopathy persists [73]. In comparison, expressing the slow Wallerian degeneration (*WldS*) allele in models of inducible glaucoma confers protection of RGC somata and slows axonopathy [71]. (Wallerian degeneration refers to gradual disintegration of axons and their myelin sheaths, which proceeds in the distal-to-proximal direction in early induced RGC axonopathy [71].) In both models, the effects depend on the RGC subtype [71].

In glaucoma and other neurodegenerative diseases, certain resident neurons become susceptible to disease damage and degeneration [74]. Important questions in neurodegenerative disease research include how, when, and why certain cells become susceptible to injury and death. Cell-specific mechanisms related to early features of disease damage, for example challenges to axonal physiology, active transport, and ultrastructure, may relate to the question of cellular susceptibility. When answered, this question could dramatically improve our ability to stop or slow neurodegeneration and directly protect certain susceptible neurons in a targeted way [71]. Therefore, it is important to consider whether factors related to the health/maintenance or degeneration of the RGC dendrites, somata, and/or axons differ across RGC subtypes.

Overall, more research is needed to determine whether the optimal approach to neuroprotection should be aimed at protecting the dendrites, the soma, the axon, or a combination. In addition, we need to determine whether this approach should differ according to the stage or severity of the neurodegenerative disease.

##### Complement: a single pathway for development, repair, and disease

Complement system research dates back to the nineteenth century, but its promise and progress continue to grow. The complement system plays context-dependent roles in immunity and development, and its pathways are implicated in a variety of clinical conditions, including autoimmune diseases, ischemia–reperfusion injury, and neurodegeneration [63, 75, 76]. During development and aging, astrocytes and microglia support the formation, pruning, and remodeling of neuronal synapses via the complement pathway [77, 78]. In a system of neuron-glia crosstalk, receptors at neuronal synapses direct the actions of glia. Through the expression of C1q, C3, or phosphatidylserine, synapses are tagged for elimination during discrete developmental windows [79, 80]. However, complement-mediated synapse elimination can become aberrantly reactivated and become part of the pathology underlying neurodegeneration. The complement system is one of several neuroimmune systems that have proven to be general mechanisms for pathological synapse loss in multiple neurodegenerative diseases, including glaucoma [79], Alzheimer’s Disease (AD) [81], multiple sclerosis (MS) [82, 83], and Huntington’s disease (HD) [63, 70]. The complement system is therefore an important candidate target for potential neuroprotection with temporal specificity. 

Recent research focused on models of Huntington's Disease (HD) to study the complement system while avoiding the heterogeneity typical of most neurodegenerative diseases [63]. This research revealed that complement system mechanisms mediate the selective, early degeneration of cortico-striatal synapses, including using a combination of HD mouse models, and HD human brain tissue and cerebrospinal fluid (CSF). The outcomes of the study demonstrated that blocking the complement system in HD mice may be an early therapeutic approach to preventing or slowing disease-associated cognitive changes. Future directions will include applying this research in humanized disease models, followed by appropriate functional testing outcomes [63], and expanding the analyses of CSF from longitudinal cohorts of HD and across other neurodegenerative diseases (namely AD). This promising research will be applied to other models of neurodegenerative disease, although disease heterogeneity will present a challenge [84]. Future directions also include elucidating the pathway dependencies on time and environment, although progress has been made in describing mechanisms of region-specific and synapse-specific degeneration [85, 86].

##### SARM1/NMNATs: a pharmacologically targetable pathway of axon self-destruction

Cellular aging and axon survival are closely linked with the biosynthesis and metabolism of nicotinamide adenine dinucleotide (NAD +), a major electron carrier for intracellular oxidation–reduction (redox) reactions. NAD + biosynthesis is catalyzed by nicotinamide mononucleotide adenylyltransferase 1 (NMNAT1) and NMNAT2. NAD + is a major regulator of energy metabolism due to its role as an essential coenzyme to the enzymes involved in fatty acid oxidation, glycolysis, and the citric acid (Krebs) cycle. Conversely, NAD + depletion promotes the pathophysiology of aging and cancer through mechanisms related to energy metabolism, mitochondrial dysfunction, inflammation, DNA damage, and circadian rhythm. Axon growth and survival depend on activity of the central enzyme in NAD biosynthesis, NMNAT2, and axons degenerate rapidly when this activity is lost [87]. Relatedly, NMNAT2 is a chaperone that aids in the refolding of misfolded proteins.

**Fig. 3:**
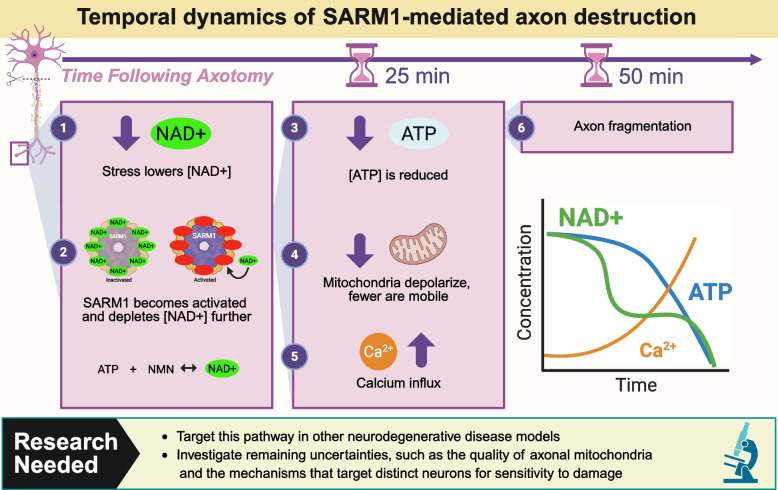
Temporal dynamics of SARM1-mediated axon destruction [69, 92]

The SARM1/NMNAT2 pathway regulates the choice between axon survival and axon degeneration. While NMNAT2 is an NAD + biosynthetic enzyme and axon survival factor [87], SARM1 is an NAD + cleaving enzyme and the central executioner of pathological axon degeneration [88]. SARM1 is activated by a variety of pro-degenerative stimuli including mitochondrial dysfunction, axon transport defects, neuroinflammation, or metabolic dysfunction. Once activated, SARM1 induces a dramatic drop in NAD +, thereby triggering a metabolic catastrophe that results in a stereotyped axon destruction program that leads to axon fragmentation within a few hours [89] (Fig. 3). The discovery that SARM1 is an enzyme led to the development of both small molecules and gene therapeutics that can inhibit SARM1 enzymatic function and thereby protect injured axons [90, 91].

Overexpression of NMNAT2, as well as the closely related NMNAT1/Wlds and loss-of-function of SARM1, provide profound neuroprotection in multiple models of neurodegeneration [68, 92, 93]. SARM1 Knockout (KO) inhibits neurodegeneration in models of traumatic axon injury, traumatic brain injury, chemotherapy/metabolic/genetic peripheral neuropathies, and retinal degeneration [94, 95]. Importantly, there is now strong evidence that the NMNAT2/SARM1 pathway is a key driver of degeneration in glaucoma. NMNAT2 is downregulated in glaucomatous retina ganglion cells (RGCs) and overexpression of NMNAT2 in RGCs rescues glaucomatous neurodegeneration [96]. SARM1 induces RGC axon and cell body loss in an inflammatory TNF model of glaucoma [69], and a mouse model of silicone-oil-induced ocular hypertension [68]. In both cases, SARM1 loss leads to protection of both RGC soma and axons. In a model of nonarteritic anterior ischemic optic neuropathy (NAION), SARM1 loss in combination with CNTF treatment provides synergistic, long-term protection of RGCs from death [97]. These findings suggest that SARM1 inhibition may be a promising treatment for optic neuropathies and diseases of RGC loss. Interestingly, SARM1 inhibitors are now in clinical trials [94], raising the hope that the efficacy of targeting SARM1 for neuroprotection will soon be tested in patients.

##### Candidate targets for RGC neuroprotection

In addition to the complement and SARM1/NMNATs pathways, recent progress has been made in identifying other potential targets for neuroprotection (Table 2). Further investigating the therapeutic potential of these targets will require differentiating their intrinsic and extrinsic mechanisms of action. Determining the optimal therapeutic strategy will also involve evaluating the efficacy of therapeutic approaches to somal protection versus approaches to axon regeneration. A major challenge will be determining optimal drug combinations and synergies.

**Table 2:** Recent potential drug-target candidates for glaucoma

Target	Reported Outcome(s)	Select Literature
ANXA2 (ILK pathway)	Neuroprotection and axon regeneration	[98]
Mpp1 (ILK pathway)	Neuroprotection and axon regeneration	[98]
Osteopontin	Neuroprotection	[99]
Osteopontin/Spp1	Neuroprotection in SOHU model (not ONC)	[100]
Osteopontin/Spp1 + IGF-1	Neuroprotection in ONC	[101]
Spp1 (ILK pathway)	Axon regeneration	[98]
Plin2	Axon regeneration	[98]
Numb1	Neuroprotection	[37]
Galectin-1 (ILK pathway)	Axon regeneration	[98]
Galectin-3	Neuroprotection	[102]
PTEN	Neuroprotection and axon regeneration	[103]
SOCS3	Neuroprotection and axon regeneration	[103]
CNTF (AAV2-CNTF only) - Mediated by CCL5	Neuroprotection and axon regeneration	[103, 104]
PTEN + SOCS3 + CNTF	Neuroprotection and axon regeneration (synergistic efficacy)	[103]
DLK/JUN	Neuroprotection (synergistic efficacy)	[31]
Lipoxins	Neuroprotection	[105, 106]
ATF/CHOP	Neuroprotection	[96]
TRAK1	Neuroprotection	[100]
KIF5B	Neuroprotection	[100]
mOPTN + TRAK1 + KIF5B	Neuroprotection (synergistic efficacy)	[100]
CaMKII T286D	Neuroprotection	[107]
Oncomodulin + CPT-cAMP + SDF-1	RGC neuroprotection and axon regeneration (glaucoma); Peripheral nerve, spinal cord axon regeneration (peripheral nerve injury)	[108] [109]
*Bax-/-*	RGC soma protection (axonopathy persists)	[73]
*Wld*^*s*^	RGC soma protection, slows axonopathy	[71]

AAV2, Adeno-associated virus type 2; ANXA2, Annexin A2; ATF, Activating Transcription Factor; *Bax-/-*, Bcl2-associated X protein; CHOP, CCAAT-Enhancer-Binding Protein Homologous Protein; CaMKII T286D, Calcium/Calmodulin-Dependent Protein Kinase II, T286D mutation; CCL5, Chemokine (C–C motif) Ligand 5; CNTF, Ciliary Neurotrophic Factor; CPT-cAMP, Cyclic AMP analogue; DLK, Dual Leucine Zipper Kinase/Jun; IGF-1, Insulin-Like Growth Factor 1; ILK, Integrin-linked kinase; KIF5B, Kinesin Family Member 5B; mOPTN, (mouse) Optineurin; Mpp1, Matrix Metallopeptidase 1; ONC, optic nerve crush; Plin2, Perilipin 2; PTEN, Phosphatase and Tensin Homolog; RGC, retinal ganglion cell; SDF-1, Stromal Cell-Derived Factor 1; SOCS3, Suppressor of Cytokine Signaling 3; SOHU, silicone oil-induced ocular hypertension underdetected; Spp1, Secreted Phosphoprotein 1; TRAK1, Trafficking Kinesin Protein 1; *Wld*^*s*^, expression of the slow Wallerian degeneration (WldS) allele

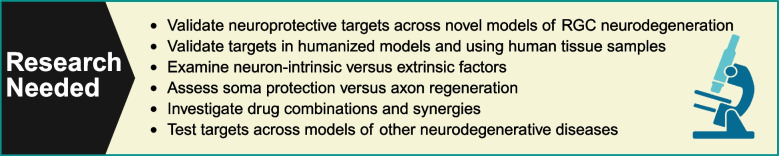

#### A transplant approach to RGC neuroprotection

Another approach to RGC neuroprotection involves transplanting neural stem cells (NSCs) into the retina. This new approach aims to protect existing RGCs from neurodegeneration, as NSCs secrete neurotrophic factors that promote RGC survival and regeneration [110].

One recent study saw success after transplanting human NSCs at the site of injury in the rat optic nerve crush model of glaucoma. There was a modest improvement in RGC survival and axon regeneration. NSCs were able to integrate with the injured optic nerve, suggesting they may aid in functional connectivity. NSC transplantation is likely to be tested in other models of glaucoma, and future studies should include functional outcome measures [110].

### New models of RGC neurodegeneration

**Fig. 4:**
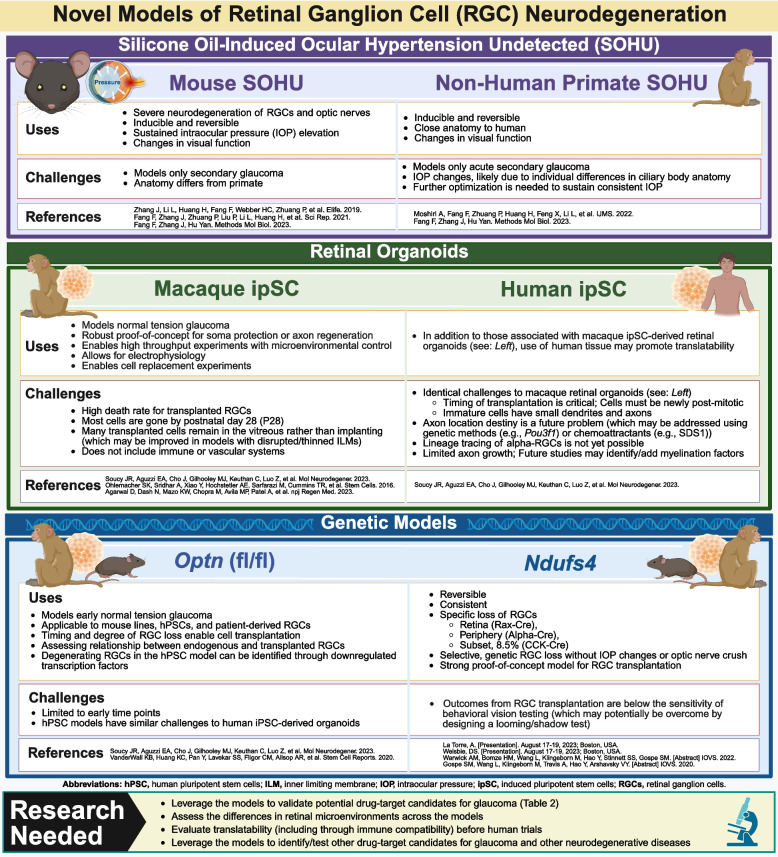
Novel models of retinal ganglion cell (RGC) neurodegeneration [114–119]

Many neurodegenerative disease models are now available to test the efficacy of new drug candidates including new models specifically developed to address concerns with translatability. Examples include the novel models of glaucoma detailed in Fig. 4. As more disease models and drug candidates become available, it will remain critical to identify the experimental endpoints suited to each situation. For example, it may be appropriate to add behavioral vision testing when evaluating the success of a new therapy for glaucoma. Other commonly used models of glaucoma include the DBA/2J mouse and optic nerve crush [111, 112]. The advantages and disadvantages of other glaucoma models are detailed in Evangelho, Mastronardi, & de-la-Torre [113].

## Strategies for pan-neurodegenerative disease therapies

### Unmet clinical need for neurodegenerative diseases

While the heterogeneity, pleiotropy, and complexity of neurodegenerative diseases have inhibited the development of effective therapies to date, investigating commonalities shared by different neurodegenerative diseases might enable the development of pan-neurodegenerative therapies. The white paper report from “Solving Neurodegeneration” 2021 suggested that specific strategies for developing pan-neurodegenerative disease therapies could arise from investigating critical underlying mechanistic features of neurodegeneration [11]; For example, they proposed investigating the role of environmental factors, neuroinflammation, metabolic stress, neurovascular coupling, and genetics across different neurodegenerative diseases [11]. To this end, the Glaucoma Research Foundation formed Catalyst for a Cure Team 4 (CFC4) in 2022 to explore the commonalities between glaucoma and other neurodegenerative diseases.

### Approach to developing pan-neurodegenerative disease therapies

The development of highly effective cancer therapies was made possible by decades of research into the tumor microenvironment and the role of the immune system in cancer [1, 120]. Similarly, neurodegenerative disease researchers are focusing on the neurodegenerative disease microenvironment and the role of immunity in an attempt to identify new opportunities for treatments.

This review summarizes recent progress in these research areas, borrowing the framework from cancer research and oncology that focuses on the roles of the disease microenvironment and immunity. The concept of pan-disease treatments has proven utility in oncology, where chemotherapy, radiation, immunotherapies, and certain other targeted therapies can be prescribed across different cancer indications. In the United States, these and other advances in cancer treatment have resulted in a continuous decline in cancer mortality since 1991, including improved survival rates for some of the most common types of cancer [1]. Meanwhile, the public health crisis posed by neurodegenerative diseases is becoming increasingly urgent as the population continues aging [4, 5]. Catalyst for a Cure Team 4 is a collaboration between researchers with expertise in neurodegenerative disease, cancer and bioinformatics who are leveraging advanced and adapted technologies toward identifying targets for future pan-neurodegenerative disease therapies.

### Adapting transcriptomics to study the microenvironment and immunity

**Fig. 5:**
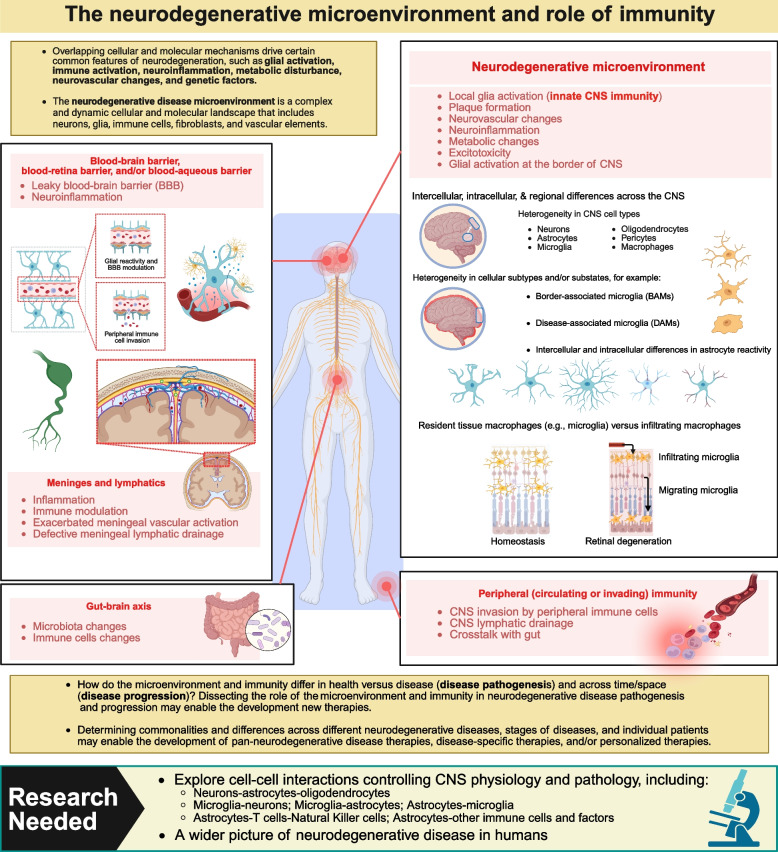
The neurodegenerative microenvironment and role of immunity [124–130]

The neurodegenerative disease microenvironment is a complex and dynamic cellular and molecular landscape that includes neurons, glia, immune cells, fibroblasts, endothelial cells, and vascular elements. Overlapping cellular and molecular mechanisms drive certain common features of neurodegeneration, such as glial activation, immune activation, neuroinflammation, metabolic disturbance, neurovascular changes, and genetic factors [11, 121, 122] (Fig. 5). Deeper knowledge of these concepts will likely lead to the development of new and more efficacious investigative therapies, such as precision-targeted treatments against disease-specific features of the immune response or the microenvironment [123]. Identifying therapeutic targets within these shared mechanisms also represents potential opportunities to develop pan-neurodegenerative therapies to alleviate the progression and severity of multiple neurodegenerative diseases [11, 22, 122].

One approach to uncovering these mechanisms involves transcriptomic analysis using single-cell or single-nuclei RNA sequencing (sc/snRNAseq). Among other applications, these tools are particularly useful for identifying pathological substates of reactive astrocytes and microglia from heterogeneous populations in models of neurodegeneration. Limitations to scRNAseq include unreliability or imprecision in detecting genes expressed at low levels. Two additional factors limiting widespread use of sc/snRNAseq are cost as a barrier to entry, and the complexity of data analysis limiting data mining. The cost and availability of scRNAseq are likely to improve as it becomes an essential tool for biomedical research [131]. The rising number of sc/snRNAseq technologies available for use is also driving competition and leading to decreased consumer costs [132, 133]. Artificial intelligence is also increasing the accessibility of sc/snRNAseq by aiding and improving data analysis [134].

Novel adaptations of scRNAseq now allow for specific uses in vitro*, *in vivo*,* and in situ (Table 3). Critical cell–cell interactions will likely be revealed by applying an array of these techniques, providing potential opportunities for precision therapies and pan-neurodegenerative treatments. Additionally, in situ methods may allow for localizing cell–cell interactions of interest [131]. Subcellular localization of pathological features may provide insight into the sensitivity of axons to damage compared with their somata. Such knowledge might also explain the susceptibility of peripheral RGC axons over foveal RGC axons in glaucoma, or the susceptibility of motor neurons over sensory neurons in peripheral neuropathies. Importantly, transcriptomic analysis of human tissue samples and humanized models will be necessary to develop translatable targeted therapies. Future studies are likely to use scRNAseq to address other research challenges, as summarized in Table 4 [139].

**Table 3:** Adapted RNASeq for cell–cell interrogation in the CNS

RNASeq	Use	Example	Challenge	Literature
FIND-Seq	Sort cell subsets of interest based on nucleic acid expression (mRNA or viral DNA)	Detect rare astrocyte or T-cell subsets of interest	Limited to frozen or fresh samples (not fixed)	[135] [136]
RABID-Seq	Reconstruct cell–cell interactions in vivo or in clinical samples	Study cell–cell interactions	-	[137, 138]
TA-RABID-Seq	Define cell–cell interactions in the tumor microenvironment	Humanized animal models	-	[138, 139]
SPEAC-Seq	Communication between cells of choice (co-cultured cells; a library, and a reporter)	Rule out false positives and false negatives	Limited to in vitro	[140]
MAP-Seq	Translating transcriptomics into projection maps, “projectomics”	Physically map cell connections	Postsynaptic targets are not identified	[141]
Trans-Seq	Translating transcriptomics into connection maps, “connectomics”	Functionally connect cells; Identify post-synaptic circuits from genetically defined presynaptic neurons	-	[142]

CNS, central nervous system; FIND-Seq, Fast Identification of Nuclei using DNA barcodes Sequencing; MAP-Seq, Multiplexed Analysis of Projections by Sequencing; RABID-Seq, Rabies Barcode Interaction Detection followed by Sequencing; TA-RABID-Seq, Tumor-Associated RABID-Seq; SPEAC-Seq, Systematic Perturbation of Encapsulated Associated Cells followed by Sequencing

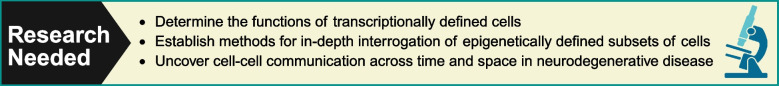

**Table 4:** Challenges and solutions for studying cell interactions

Challenges	Proposed Solutions
*How can we study rare cells?*	FIND-Seq; Pre-enrich for cell type/substate of interest (e.g., reporter mouse lines, FACS/FANS enrichment)
*Are transcriptomically distinct cells functionally distinct?*	Test function using high-quality, multicellular in vitro platforms; Recapitulate transcriptomic profiles (from tissue data) in vitro by applying a “cocktail” of small molecules and transcription factors [143]; Perform in vivo Perturb-seq experiments, as described in Wheeler et al. [140]
*How can we identify cell–cell interaction mechanisms?*	SPEAC-seq; RABID-seq
*What is the function of cell–cell interactions?*	CRISPR Functional Validation; High-quality, multicellular in vitro platforms
*Where do these interactions occur?*	In situ (spatial) transcriptomics and epigenetics [131]
*How can we target cell–cell interactions of interest?*	Small molecule antibodies; [137, 144] Engineered microorganisms [145, 146]

CRISPR, Clustered Regularly Interspaced Short Palindromic Repeats; FACS, Fluorescence-Activated Cell Sorting; FANS, Fluorescence-Activated Nuclear Sorting; FIND-Seq, Fast Identification of Nuclei using DNA barcodes Sequencing; RABID-Seq, Rabies Barcode Interaction Detection followed by Sequencing; SPEAC-Seq, Systematic Perturbation of Encapsulated Associated Cells followed by Sequencing

#### Challenges and future directions

Numerous sc/snRNAseq datasets have been published from studies on healthy eye and brain tissue [147–152], AD-affected human and animal brains [153–155], and mouse eyes with glaucoma (DBA/2J model) or optic nerve injury [148, 156, 157]. Fewer datasets are available from human tissue with glaucoma or glioma [158, 159]. The available neurodegenerative disease datasets are generally limited to later stages of the disease, excluding early-stage information and failing to represent temporal changes in disease mechanisms. At the snRNAseq level, they can be underpowered for low-abundance cell types, or poorly powered for microglia, astrocytes, and endothelial cells [160]. The datasets can also lack notice of severity across diseases. To study the common mechanisms underlying the onset and progression of neurodegeneration, future studies must consider time and other contextual factors related to disease severity. Creating new human datasets will be crucial for translating an RNAseq-based approach into viable human drug targets. To fill this knowledge gap, researchers are creating novel datasets like the multi-omics atlas of the human retina [161].

However, developing new RNAseq datasets poses significant challenges, such as establishing reliable single-cell isolation procedures that do not alter the transcriptome of immune cells [162] or underpower individual cell types. In general, studying cellular heterogeneity at the transcriptomic level remains difficult because low-abundance cell types are often underrepresented. For example, while sc/snRNASeq datasets from AD tissue are beneficial for studying neurons and oligodendrocytes, they often lack sufficient data on other CNS cells like microglia, astrocytes, and endothelial cells. To address this issue, recent studies use single-cell or single-nuclei sorting to enrich rare cell types for sequencing while maintaining oligodendrocyte capture rates and depleting neurons [160, 163]. However, the current state-of-the-art methods for separating retinal cell types still face difficulties isolating RGCs, distinct RGC subtypes, astrocytes and other retinal cell types [164].

Low concordance between datasets can also present a challenge, and may be reflective of pathological variability or underlying genetic variance among tissue samples [160, 163]. Recent studies aim to resolve this issue by including sample populations that were underrepresented in previous studies, such as populations of older age or a certain genotype [160, 163].

### Progress in pan-disease neuroprotection

#### Catalyst for a Cure Team 4

**Fig. 6:**
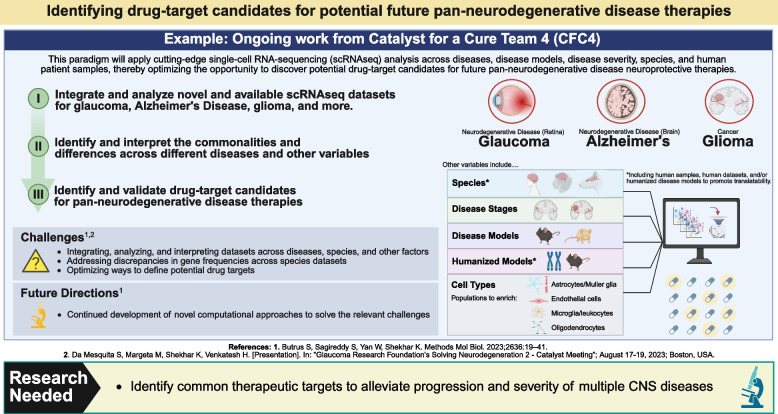
Novel paradigm for identifying pan-neurodegenerative disease drug targets [164, 168]

The Catalyst for a Cure Team 4 (CFC4) is using scRNAseq to investigate and compare the neurodegenerative microenvironments of AD, glaucoma, and glioma (brain tumors associated with secondary neurodegeneration) [165–167] in hopes of identifying pan-neurodegenerative drug targets. Their approach involves identifying, validating, and investigating common cell types and pathways across CNS pathologies (Fig. 6). This novel strategy is made possible by the increasing number of publicly available transcriptomic datasets, including for healthy and diseased cells, tissues, organs, and species. The team is also closing knowledge gaps by creating new datasets from animal models and human tissue samples of AD, glaucoma, and glioma. Overall, this approach creatively dissects the microenvironment while accounting for temporality, disease severity, and other challenges currently experienced across the field. This strategy of validating targets by testing across species, diseases, and disease models will likely aid in the translatability of potential pan-neurodegenerative therapeutics [164].

##### Challenges and future directions

CFC4 is working on addressing the current challenges associated with developing new RNAseq datasets, including those discussed in Adapting transcriptomics to study the microenvironment and immunity. The team have begun their analyses with post-mortem tissue samples from the posterior segments of mice and humans, namely the retina and optic nerve. The samples include healthy mice and mice with induced RGC neurodegeneration (microbead model and optic nerve crush model [102, 169]). The post-mortem human tissue samples include from healthy eyes and glaucomatous eyes. They are establishing reliable procedures for single-cell isolation that respect individual cell types and do not alter the transcriptome of immune cells, including by optimizing their non-neuronal cell enrichment strategies and cell isolation procedures [12, 169, 170].

The approach also poses challenges related to integrating and analyzing data sets across CNS diseases, species, and other factors of variability, while addressing discrepancies in gene frequencies across species datasets, and optimizing ways to define potential drug targets [12, 168]. Integrating these datasets requires the continued development of novel computational approaches [103, 148, 151, 156, 168, 171]. CFC4 is therefore developing new procedures and computational approaches for analyzing each of the datasets individually and integrating them. They are bringing together the results of this multisite initiative to create a comprehensive immune cell atlas of the ocular posterior segment.

After identifying common pathways, the CFC4 team will uncover their functional significance and test their suitability as drug targets, while also considering the persisting challenges in humanizing disease models and increasing the translatability of potential therapeutics [164]. In an RNAseq-based approach, researchers can address these challenges by including human datasets in their analyses. Further, new animal models can be created using genetic approaches to deliver human versions of the gene of interest. Human induced pluripotent stem cells (iPSC) assays are also an option. With the advent of new models, appropriate outcome measures should be considered, such as functional assessments. In addition to using human tissue samples and existing human RNAseq datasets, CFC4 are validating their analyses from animal models using human proteomics and integrating their analysis with datasets from iPSC models [164]. Additionally, CFC4 are optimizing their multi-method approach to resolve functional information from the RNAseq datasets, including using CellPhoneDB analysis (for receptor/ligand interactions) [172], and comparing the results across the different disease pathologies. To date, this level of work has included assessing and comparing datasets from human glaucoma, mouse RGC neurodegeneration, human glioma, and models of glioma.

To further analyze the functional significance of their RNAseq analyses, CFC4 are optimizing a pipeline for achieving spatially resolved transcriptomics from the various tissue types (including brain, retina, and optic nerve) using MERFISH [173] and additional methods as needed, such as immunohistochemistry (IHC), slide-seq (snRNAseq) with spatial reconstruction, or high-resolution optical coherence tomography (OCT). For example, IHC can be used to add spatial information to differentially expressed genes (DEGs) identified during the RNAseq analyses. IHC, for example, can also be used to visualize cell-specific spatial boundaries in the optic nerve, or by differentially labeling resident macrophages versus infiltrating peripheral monocytes in the retina. This level of work is also being supported by longitudinal RNAseq with lineage tracing to map the migration of resident monocyte populations in the retina [164].

Further, the team plans to explore specific questions related to the functional significance of their RNAseq analyses. For example, they are specifically assessing the global role of microglia in neuronal excitability. The current understanding is that neurons become increasingly hyperexcitable over time in models of glioma [174, 175] and AD [176]. In glaucoma models, RGC excitability is transiently increased only in early stages of the disease [177]. CFC4 is therefore using their various models of glaucoma, glioma, and AD to grow our knowledge of the relationship between microglial activity and the relative excitability of neurons in different diseases and across stages of disease.

#### Potential drug-target candidates

There has been progress identifying candidate drug-targets for potential pan-disease neuroprotective therapies (Table 5). Future studies will likely validate and test these targets using various models of neurodegenerative diseases, and including humanized models and human samples.

**Table 5:** Potential drug-target candidates for pan-disease neuroprotection

Target	Reported Outcome(s)	Select Literature
Galectin-3	Neuroprotection	[178–182]
APOE	Neuroprotection	[102]
GCK-IV Kinase	Neuroprotection and axon regeneration	[183]
Complement	Neuroplasticity and neuroprotection	[63, 66]
SARM1/NMNAT	Neuroprotection and axon regeneration	[68–70]
NAD +	Neuroprotection and axon regeneration	[68, 184, 185]
DLK - Protective downstream kinases include LZK, MKK4/7, and JNK1-3 - Protective transcription factors include MEF2A, SOX11	Neuroprotection	[186, 187]
CD44	Neuroplasticity and anti-inflammatory	[188]

APOE, Apolipoprotein E; GCK-IV Kinase, Glucokinase IV; SARM1/NMAT, Sterile alpha and Toll/interleukin receptor motif-containing 1/nicotinamide-nucleotide adenylyltransferase; NAD +, nicotinamide adenine dinucleotide; DLK, Dual leucine zipper kinase; LZK, Leucine zipper kinase; MKK4/7, mitogen-activated protein kinase kinases 4 and 7; JNK1-3, c-Jun N-Terminal Kinases 1–3; MEF2A, Myocyte Enhancer Factor 2 A; SOX11, SRY (sex determining region Y)-box 11

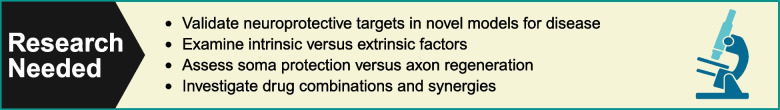

### Glia in the neurodegenerative microenvironment

**Fig. 7:**
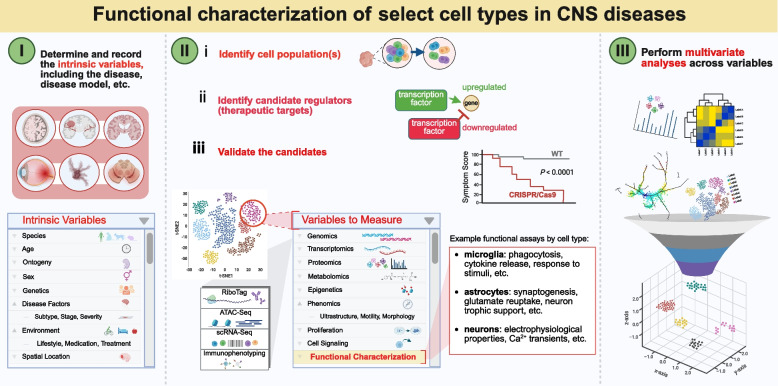
Approach to functional characterization of select cell types in CNS diseases [139]

Glia are present in high numbers throughout the brain, spinal cord, and retina, and they undergo a multitude of heterogeneous and phenotypic changes during disease [189, 190]. Glial cells are key components of the neurodegenerative disease microenvironment, and a recent focus of neurodegenerative disease research is defining the role of specific glial cell types and subtypes in responding and contributing to neurodegenerative disease [127, 190] (Fig. 7). Studies investigating glia have the potential to provide key insights into neurodegenerative disease pathogenesis and progression, as well as potential therapeutic opportunities, such as glia-targeting approaches to achieving neuroprotection.

This microenvironment-based perspective for exploring potential therapeutic opportunities is directly informing the work of CFC3 and CFC4. CFC3 is focused on identifying and validating neuroprotective drug targets for retinal ganglion cells in glaucoma, and the proposed targets span both neuron-intrinsic mechanisms and neuron-extrinsic mechanisms (e.g., involving microglia, astrocytes, and/or other non-neuronal cells), many of which relate directly to microenvironmental interactions. Next steps include validating these targets across disease models and comparing the efficacy of neuron-intrinsic versus neuron-extrinsic approaches—an effort that hinges on understanding the contribution of glia to RGC neurodegeneration and protection. CFC4, in parallel, is searching for pan-disease neuroprotective strategies, an approach that enables the assessment of both intrinsic and extrinsic cellular drivers of neurodegeneration, including glial and immune cell responses. Together, these efforts aim to map the neurodegenerative microenvironment in order to identify shared mechanisms and guide the development of targeted and pan-disease treatments.

#### Microglia in the microenvironment

The ongoing work of CFC 3 and 4 includes investigations of potential drug-target candidates related to microglia, such as the complement pathway, Galectin-3, APOE, or certain spatially defined subpopulations of microglia, such as border-associated microglia (BAMs).

##### Physiological microglia in the healthy CNS

Much of the current literature on neuroprotection focuses on microglia, an abundant and dynamic glial cell type that responds to its environment and actively signals to neurons and other cells [191]. Microglia support normal development and contribute to cellular aging, including pruning and remodeling CNS synapses [75, 84, 192].

##### Reactive microglia in CNS pathologies

During disease, microglia are recruited to sites of pathology and become induced into one of several reactive states, each with unique morphology, gene expression, and functions. These reactive microglia states, and their evolution over time, have been well described in several neurodegenerative disease contexts [155, 193–196]. Microglia and astrocyte reactivity occur coincidentally across the prodromal, early, and late stages of neurodegeneration.

##### Challenges and future directions

As microglia display unique gene expression profiles in response to distinct pathological insults, the role of microglia in disease can be first studied using longitudinal and spatial transcriptomics before proceeding to functionally characterize distinct, transcriptomically defined substates of reactive microglia. Single-cell/single-nucleus RNAseq and bulk RNAseq have revealed that reactive microglia in glaucoma upregulate *Gals* (the gene encoding Galectin-3) [102, 181], which is associated with inflammation and implicated in retinal degeneration [197], Alzheimer's Disease [180], Huntington’s [179], amyotrophic lateral sclerosis [182] and multiple sclerosis [178]. Small molecule inhibitors targeting Galectin-3 have proven neuroprotective in multiple models of RGC injury, including optic nerve crush and glaucoma [102, 181]. 

Complicating the understanding of reactive substates of microglia and astrocytes is that many markers are expressed across the two cell types. An example of this is *APOE*, which is highly expressed by astrocytes in a physiological state, but upregulated in microglia during reactivity. APOE regulates Galectin-3 and *APOE4* is a major risk factor for Alzheimer’s disease but is associated with a reduced risk of glaucoma [102]. The underlying mechanisms behind this specificity still need to be elucidated, further emphasizing the importance of studying the disease microenvironment in the context of cell-type specific functional changes. Uncovering the unique functions of disease-associated reactive microglia, and astrocytes, involves investigating the downstream functional effects of altered gene expression profiles [191, 198].

#### Astrocytes in the microenvironment

The work of CFC 3 and 4 includes exploring the role of astrocytes in disease pathogenesis and potential opportunities for neuroprotection, including investigations into the complement pathway, lipoxins, or the role of astrocytes in recruiting peripheral immune cells to sites of CNS damage. There are challenges and potential opportunities that remain which are specific to astrocytes within the neurodegenerative disease microenvironment.

##### Physiological astrocyte subtypes in the healthy CNS

Astrocytes are abundant CNS glia that provide vital homeostatic support for neurons, microglia, and many other CNS cells [199]. They perform an array of functions in response to diverse molecular signals, including from neurons, glia, and blood-borne molecules or immune cells [200, 201]. Numerous physiological “subtypes” of astrocytes are present in the healthy CNS, and these subtypes exhibit diversity in structure, genetics, and (possibly) function [201].

The physiological subtype of astrocytes is determined by developmental programs and factors such as anatomical location, genetic predisposition, and aging [130, 201]. 

Astrocytes vary across regions of the CNS [130] as different regions require specific astrocyte support. For instance, dopaminergic neurons in the striatum are supported by astrocytes that express a distinct set of genes (e.g., *Crym*) [202]. Astrocytes support the structural integrity of blood–brain barrier (BBB), aid in synapse formation and support synapse stabilization and function, recycle ions and neurotransmitters, maintain low extracellular neurotransmitter concentrations, produce neurotrophic factors, and provide metabolic support [139]. Astrocytes also contribute to synapse pruning and phagocytosis of cellular debris during normal development [66, 203–205]. Astrocytes play a critical role in various other CNS processes, including neurogenesis, neuroplasticity, neuronal activity, and cognitive and behavioral brain function.

##### Reactive astrocytes substates in CNS pathologies

Astrocytes are early responders to CNS trauma, infection, and disease-associated stimuli [121]. They rapidly respond to local pathological factors through "reactivity," an early hallmark in neurodegenerative diseases such as glaucoma, Alzheimer's, and Parkinson's [121, 201]. The process of induction to produce reactive astrocytes includes diverse changes in morphology, gene expression, and function, driven by CNS pathologies and subsequent, stimulus-induced responses like cell–cell interactions and immune signaling [121, 201, 206]. For instance, during the onset or progression of neurodegenerative diseases, astrocytes react to local increases in proinflammatory cytokines, reactive oxygen species, or infiltrating immune cells, like T cells [139].

Reactive astrocyte “substates” vary widely based on CNS insult type, severity, anatomical location, genetic factors, and aging [201, 207]. Regional differences in astrocytes can be particularly evident during aging or disease [207], such as in Huntington's Disease, which primarily affects the striatum (e.g., GFAP + and S100B + astrocytes) [208]. Critical evidence indicates that striatal astrocyte reactivity contributes to cognitive and behavioral changes in HD (in part because specific physiological astrocytes modulate neural activity in the healthy striatum) [121]. Parkison’s Disease primarily affects the basal ganglia, including the striatum. Recent evidence suggests that striatal astrocytes may affect movement deficits in mice with hemi-parkinsonism [209]. Distinct substates of reactive astrocytes have also been identified in human tissues with neurodegenerative disease, including Alzheimer’s Disease [163, 210]. The heterogeneity of reactive astrocyte substates is highly complex at the transcriptomic, epigenetic, and functional levels [121]. While researchers predict that astrocyte functions vary according to distinct reactive states, few substate phenotypes have been validated at the functional level [211]. There is considerable focus in the field on continuing these characterizations [211].

##### Challenges and future directions

It is evident that reactive astrocytes display broad functional diversity, with some contributing directly to neurotoxicity by increasing extracellular concentrations of neurotoxic molecules, decreasing their support for neurons, or pruning neuronal synapses as part of the degenerative process [66, 105, 203–205]. Others may indirectly contribute to neurotoxicity through interactions with microglia or immune cells, sometimes creating a feedback loop of astrocyte reactivation [212–222, 222–229] (Fig. 5). Some disease-associated reactive astrocytes may also recruit and activate macrophages, contributing to CNS inflammation [206, 215]. Other substates of reactive astrocytes re-engage the cell cycle and produce a scar that borders and corrals damage lesions, such as those present following stroke, spinal cord injury, or other acute, physical injuries to neurons [128, 230–233]. Through these and other mechanisms, reactive astrocytes can both promote and suppress CNS pathology. While we know that astrocyte functions are context-dependent and localized, and that factors like genetics, disease, and environment influence the net effect of reactive astrocytes on disease progression, it is crucial for understanding neurodegenerative disease onset and progression to learn how reactive astrocyte functions are regulated over time and across different regions of the CNS.

**Fig. 8:**
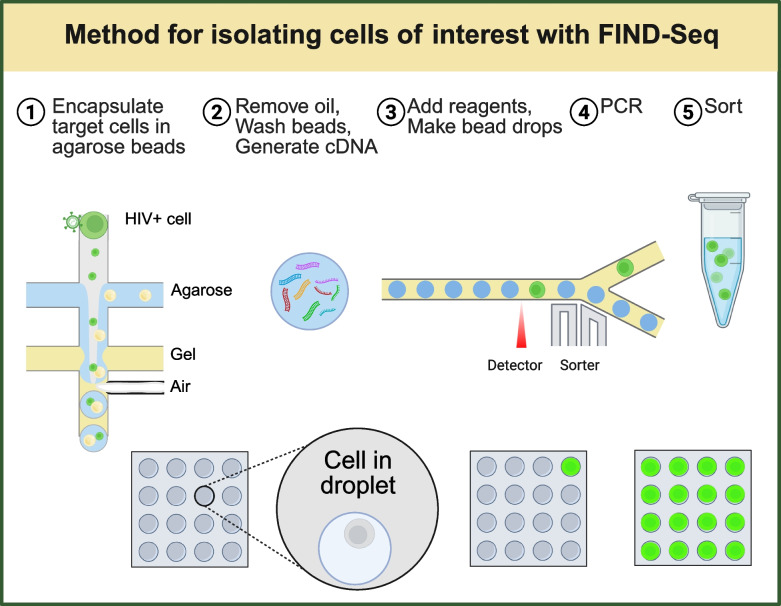
Method for isolating cells of interest with FIND-Seq [139]

Astrocyte heterogeneity further extends to substates that respond to inflammation in distinct ways [139]. Distinct substates of inflammation-responsive reactive astrocytes have been identified in many human neurodegenerative diseases. Their phenotypic differences are being investigated using FINDseq (Focused Interrogation of cells by Nucleic acid Detection and Sequencing), a technique for detailed genomic, transcriptomic, and proteomic analysis that detects rare astrocyte phenotypes [135, 136, 139] (Fig. 8). However, additional research is needed to identify and characterize the functions of different inflammatory reactive astrocyte substates. Recent studies suggest some inflammatory reactive astrocyte substates are spatially distinct, such as those near invading immune cells, pathogenic protein plaques (e.g., amyloid, alpha synuclein), tumors, or the border of the CNS [143, 222]. Additionally, even within these specific niches, astrocyte functional heterogeneity should be considered. A recent study described a population of astrocytes at the border of the CNS that was induced by interactions with natural killer (NK) cells, and capable of inducing T-cell apoptosis and suppressing neuroinflammation [214]. Importantly, some human and rodent inflammatory reactive astrocyte substates display similarities at the transcriptomic, proteomic, and functional levels [139, 144, 218, 219, 221, 234–236].

This astrocyte heterogeneity also extends to subcellular specialization. It is likely that distinct astrocyte processes detect the local microenvironment and engage in crosstalk with other CNS cells. Astrocytes detect stimuli in the microenvironment and compute their responses over larger areas and longer timescales than neurons [237] (the duration of astrocyte calcium transients [signals] is ~ 10 s, while neuron action potentials last only milliseconds) [238, 239]. The formation and maintenance of neural circuitry depends on the ability of astrocytes to respond to their environments through time-dependent, synchronized activity with neurons [237]. Indeed, the importance of process-specific signaling has been recently highlighted by the study of process-specific subproteomes, and their significance in astrocyte-neuron interactions and behavior [240]. Therefore, understanding the diverse abilities of astrocytes to compute time and temporally scale their responses could be key to understanding the temporal dynamics of neurodegenerative disease pathogenesis. While this is an area of development, ongoing research aims to leverage this kind of information to investigate discrete windows of the neurodegenerative disease timeline.

One study recently characterized two major clusters of neuroprotective versus neurotoxic reactive astrocytes, with each major cluster further divisible into sub-clusters, in local optic nerve trauma [241]—a major advancement for the field in characterizing astrocyte reactive substates. For the first time a molecular handle into differentially regulating these two populations was identified, namely that neuroprotective astrocytes depended on soluble adenylate cyclase and cyclic adenosine mononucleotide (cAMP)-regulation of proliferation. Manipulating these pathways changed the relative populations of neuroprotective versus neurotoxic astrocytes, and further revealed that at least one function of neuroprotective astrocytes is to suppress neurotoxic astrocytes, suppress microglial and macrophage reactivity, and thereby promote RGC survival [241].

Overall, understanding astrocyte functional heterogeneity and regulation is crucial for developing therapies for neurodegenerative diseases. For detailed molecular mechanisms and therapeutic insights related to astrocytes in neurodegenerative diseases, recent reviews by Lee et al. [242], [131], Verkhratsky et al. [243], and Liddelow et al. [201] are recommended.

### CNS immunity

Studies from the past three decades overturned long-held historical beliefs about immunity and the central nervous system. We now know that the CNS is not inherently immune privileged, nor completely isolated from peripheral immune cells or immune factors. Rather, the role of immunity in the CNS appears two-fold: innate CNS immunity (i.e., resident, non-infiltrating immune responses) or infiltrating peripheral immunity (i.e., a relatively localized response in the CNS to infiltrating immune cells or other signals from the peripheral immune system).

Resident astrocytes and microglia express inflammatory phenotypes and drive immune-like responses in the brain parenchyma. Changes in the function of microglia, astrocytes, and endothelial cells that form the blood–brain barrier occur in response to inflammation, aging, genetics, obesity, gut dysbiosis, infection, hormone regulation, sleep deficits, and head injury [244]. Through these mechanisms, innate CNS immunity can account for the contributions of environmental risk factors and lifestyle choices toward disease. Decades of research have demonstrated critical contributions of innate CNS immune pathways in the pathogenesis of neurodegenerative diseases (Fig. 5), including AD [245–248], HD [249], and glaucoma [250]. Yet, although immunotherapies are one of the most extensively studied approaches to solving neurodegeneration, even candidates with preclinical success have shown little clinical efficacy [248, 251, 252]. However, recent studies have demonstrated potential in harnessing the innate immune response as a therapeutic strategy against neurodegeneration [244]. Here, we discuss recent findings concerning the role of inflammatory glia in the brain parenchyma, cells at the border of the CNS, and how innate immunity contributes to the breakdown of the BBB.

#### Microglia in CNS immunity

Microglia, the ‘resident immune cells of the CNS,’ can both induce and respond to reactive astrocytes and local factors associated with injury, disease, or the environment. Disease-associated microglia (DAMs) are an important hallmark of many neurodegenerative diseases (see Microglia in the microenvironment). Importantly, sustained microglial reactivity leads to a positive feedback loop with neurons that results in low-grade chronic neuroinflammation [253]. In addition to responding to local factors, new evidence indicates microglia also respond to distant injury. A recent study in a mouse model of glaucoma revealed that retinal damage induced by ocular hypertension was associated with the appearance of DAMs in distant regions of the myelinated optic nerve [36]. The underlying mechanisms and downstream consequences of this effect are unknown; however, future studies will investigate whether immune-mediated mechanisms account for this phenomenon.

Our view of CNS immunity is quickly changing as recent studies have demonstrated diverse and transcriptionally distinct CNS macrophages, particularly at interfaces such as the perivascular space, leptomeninges, choroid plexus, and dura mater. These CNS-associated macrophages (CAMs), e.g., border-associated macrophages (BAMs), include myeloid cells, lymphoid cells, and dendritic cells [254]. While it is clear they play important roles in CNS health and disease, their complexity and functions are not yet well understood [255, 256]. Understanding these cells in the healthy and degenerating brain will be critical to developing effective therapies for neuroprotection and neuroregeneration.

#### Astrocytes in CNS immunity

Astrocytes are early regulators of innate CNS immunity due to their fast responses and critical role in maintaining BBB integrity [244]. The specialized identities and functions of astrocyte subtypes can extend to distinct anatomical locations, such as *Myoc* + astrocytes, which reside on the surface of the CNS and may regulate peripheral inflammatory effects around the outermost border of the CNS [222]. Unique astrocyte substrates aggregate at sites of peripheral immune cell entry, including IFN-responsive reactive astrocytes—a population of reactive astrocytes discovered in rodents that is evolutionarily conserved in humans and coincident with neurodegenerative disease pathology [210, 221, 234]. Similarly, TRAIL + astrocytes detected at CNS borders in mice and humans counteract CNS inflammation by decreasing the proinflammatory activity of T-cells [214]. Inflammatory reactive astrocytes heterogeneity is also distinct around sites of pathology [223]. For example, studies have shown that reactive astrocytes identified in Alzheimer’s Disease postmortem tissue take on an inflammatory subtype and maintain strategic locations around plaques and other regions of pathology [143, 163, 210]. In many CNS diseases, including multiple sclerosis, astrocytes recruit and induce reactivity in peripheral myeloid cells [121, 257]. This process contributes directly to neurotoxicity and demonstrates control over oligodendrocyte processes for myelination [121, 139, 206, 215, 217]. Through this mechanism, the innate CNS immune response delineates two mechanistically distinct phases of multiple sclerosis: the relapsing–remitting phase and the progressive phase. The relapsing–remitting phase is detectable in about 85% of cases at diagnosis, and most develop the progressive phase of the disease over time [248]. Currently, therapeutics are most effective for the treatment of the relapsing–remitting phase [139]. To develop therapies for later stages of the disease, it is critical to further our understanding of the innate CNS contributions to the disease progression [139].

#### Glymphatics

Another important system–the glial lymphatic system, or glymphatic system–initially earned its name through the presumed role of astrocytes. The glymphatic system clears metabolic waste from the CNS through a network of perivascular channels formed by the astrocytic glia limitans perivascularis [258]. While the glymphatic system is not well understood, the presence of the space between blood vessels and astrocyte endfeet (the Virchow-Robin Space) has been known since the mid-1850s. The relationship to CNS immunity, however, is only recently becoming more evident. Glymphatic clearance of neurotoxic metabolites declines in animal models of Alzheimer’s Disease [259], multiple sclerosis [260], Huntington’s Disease [261], glaucoma [262], amyotrophic lateral sclerosis [263] and other neuropathologies. Therefore, future research should continue to investigate the role of CNS immunity and test its therapeutic potential. However, a wider view is needed, including the roles of the resident glymphatic system, peripheral immune cells, and other immune regulatory pathways (Fig. 5).

### Peripheral immunity

Many studies have detailed the complex, pathological involvements of the peripheral immune system in spinal cord injury [264] and peripheral neuropathies [265–267], but peripheral immunity typically receives less attention in neurodegenerative disease research [264]. However, increasing evidence shows that peripheral immunity is a critical regulator of CNS homeostasis and disease [268]. The constant crosstalk between peripheral lymphocytes and the brain-resident lymphocytes is established early-on during development, and is crucial for proper microglia maturation [269]. Importantly, the communication between peripheral, brain-border, and brain resident adaptive immune cells continues throughout the entire mammalian lifespan, and is crucial for the maintenance of CNS inflammatory responses and maintenance of neuronal activity and behavior [270–272]. However, when dysregulated, the peripheral immune system directly and indirectly contributes to the development and progression of neurodegenerative disease.

Cytokines and peripheral immune cells can extravasate into the brain parenchyma across the BBB and cause direct neurotoxicity [215]. In addition, they contribute to aberrant CNS immune responses through the activation of astrocytes and microglia (Fig. 5). Glia (particularly microglia) increase their capacity for cytokine release and decrease their support for neurons through molecular processes that may be directly linked [273–275]. This positive feedback process drives neuroinflammation and neuronal death [268]. However, there is relatively little understanding of the crosstalk between peripheral immune cells and CNS resident glia [139]. A better understanding of the role of peripheral immunity is likely to lead to treatment for the progressive phase of MS, as well as other neurodegenerative diseases.

#### The CNS meninges and lymphatic drainage

The meninges (pia mater, arachnoid mater, and dura mater) are a tri-layer tissue encasing the CNS [276]. The meninges, particularly the dura mater, contain a wide variety of resident innate and adaptive immune cells that originate directly from the blood and skull bone marrow under physiological conditions, and are distinct from CNS resident innate immune cells. Moreover, the meningeal dura harbors a lymphatic vascular system that is constantly draining CNS-derived antigens and dural immune cells, first into the peripheral cervical lymph nodes and later into the rest of the peripheral lymphatic system. This system represents a previously unappreciated direct connection between the CNS environment and the periphery [277, 278].

Meningeal lymphatics therefore participate in the process of immune surveillance of the CNS by regulating both meningeal and brain immune responses in steady state, aging, or in response to injury and disease-specific pathologies, such as MS-like demyelination or AD-like brain amyloidosis [164, 276, 279–284]. Of note, it was recently shown that the meningeal dural lymphatics extend all the way into the optic nerve, drain the posterior compartment of the eye directly into the cervical lymph nodes, and, by doing so, actively modulate immune responses within the eye [285]. Future research will focus on further exploring the role of meningeal lymphatic functions as an active regulator of the immune crosstalk between the CNS (the optic nerve and retina included) and the periphery, collectively representing a somewhat ignored vascular component with significant control over chronic inflammatory changes, the formation of deleterious neuroimmune responses, and neurodegeneration.

#### The gut-brain axis

The gut-brain axis is receiving increasing attention in the study of neurodegenerative diseases [286–289]. The gut-brain axis involves direct effects of the gut microbiome on the CNS, e.g., via microbial products that access systemic circulation before crossing the BBB and acting on resident CNS cells [290, 291]. The gut-brain axis also features indirect ways that the gut microbiome affects the CNS, e.g., via the microbial modulation of immune cells in the gut, which access the CNS and modulate the activity of resident cells [139, 214].

Targeting the gut flora represents an opportunity to develop long-term therapies for chronic inflammatory diseases known to increase the risk of neurodegeneration [7, 286–289]. These efforts may be made more effective by engineered probiotics, modified using synthetic biology approaches to produce molecules that modulate the activity of specific signaling pathways [145, 146], rather than natural probiotics, which may target pathways of interest with moderate activity while also targeting additional, unknown, signaling molecules. Research suggests gut flora directly induce transcriptional changes in peripheral immune cells, which triggers them to leave the gut and produce cytokines [145, 146, 287, 289, 292]. Similar mechanisms may explain, at least in part, how environmental risk factors (age, diet, exercise, and exposure to toxins) wreak havoc on the CNS that far overshadows the damage that could be explained by neovascularization and slow BBB breakdown. Indeed, novel platforms including zebrafish models of disease, functional screens of environmental chemical collections, genetics, and artificial intelligence open new avenues to understand the contribution effects of environmental factors to inflammation and neurodegeneration, and the mechanisms involved [139, 213, 292].

### Summary and vision for the future

**Fig. 9:**
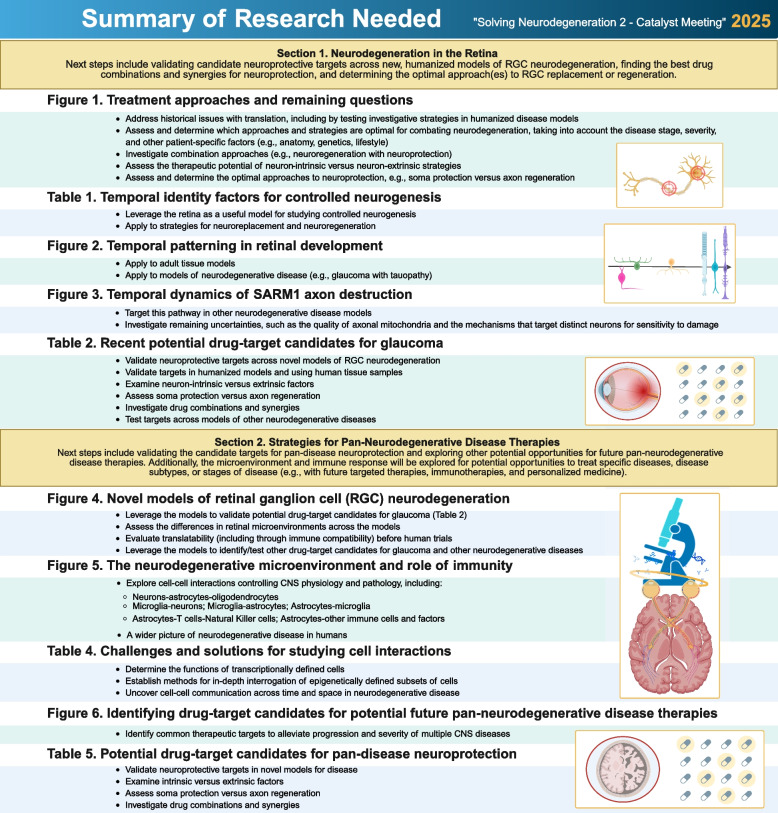
Summary of research needed (2025)

This comprehensive review resulting from the Glaucoma Research Foundation’s Solving Neurodegeneration 2: Catalyst Meeting is intended to serve as a prescriptive research aid, offering a unique focus on leveraging innovative retinal neurodegeneration models to study general mechanisms of neurodegeneration, and drawing insights from cancer research to inform the pursuit of future pan-neurodegenerative disease therapies to treat glaucoma, AD, PD, HD, MS, ALS, and/or other forms of neurodegenerative disease. Major next steps facing the field of neurodegenerative disease research include: (i) validating recently identified drug-target candidates (Tables 2 and 5); (ii) optimizing the existing investigative treatment strategies, including by investigating potential drug combinations and synergies; (iii) using humanized disease models (Fig. 4) and other strategies to address the ongoing issues with translatability; and (iv) further uncovering the roles of the neurodegenerative disease microenvironment and immunity (Fig. 5), including through creative and adaptive uses of state-of-the-art, large-scale data-driven technologies such as transcriptomics and artificial intelligence (Tables 3 and 4) (Figs. 7 and 8). Figure 9 summarizes our prescriptive suggestions for immediate actions that may expedite the development of treatments against neurodegeneration. Through the lessons and recommendations presented here, we aim to support, inspire, and mobilize researchers, clinicians, and other stakeholders to advance the management and treatment of neurodegenerative diseases.

In Strategies for pan‑neurodegenerative disease therapies, we examined a movement in neurodegenerative disease research that is focused on developing future pan-neurodegenerative disease therapies, lending inspiration from pan-disease treatments like chemotherapy and radiation which transformed clinical oncology. We also identified other lessons and concepts that the field can borrow from oncology, including disease staging, a focus on personalized medicine, and emphases on the role of immunity and the disease microenvironment. We detailed a recent collaboration between glaucoma researchers, neuroimmunologists, and cancer biologists, Catalyst for a Cure Team 4, who are leveraging their multidisciplinary expertise and advanced transcriptomics technologies in search of potential pan-neurodegenerative disease drug targets. We also discussed the related challenges in interpreting complex multivariate analyses across disease models and we proposed future research directions to address these barriers.

Looking forward, a better understanding of neurodegenerative disease pathogenesis will likely require us to further expand and piece together our knowledge of the disease microenvironment, CNS and peripheral immunity, and beyond (e.g., including complex and contemporary evolving topics such as the lymphatics system and the gut microbiome/gut-brain axis). A more holistic understanding of neurodegeneration may one day transform these devastating diseases into chronic but survivable/manageable conditions—similar to the progress made in cancer care in recent decades [1]. The future of neurodegenerative disease research and treatment might resemble today’s advanced cancer research and care, including (i) routine neurodegenerative disease screening and earlier diagnosis; (ii) biomarker testing for disease screening, diagnosis, and guiding treatment decisions; (iii) many treatment options for physicians, including pan-disease treatments, immunotherapies, and other targeted therapies; and (iv) guidelines for treatment decisions, including standard-of-care treatment regimens and recommendations for treatment sequencing.

## Conclusions

In conclusion, significant challenges persist in neurodegenerative disease research. Our ability to understand and treat neurodegeneration remains limited by the complexity of its mechanisms and its manifestation across a diverse group of heterogeneous diseases, including glaucoma, Alzheimer’s Disease, Parkinson’s Disease, and others. Therefore, one critical aspect of neurodegenerative disease research is exploration and improved understanding of the commonalities and distinctions across neurodegenerative diseases. To date, zero treatments have been approved to stop or slow neurodegeneration. Numerous monotherapy candidates have been identified but, generally, translating basic scientific discoveries into improved clinical management remains a major hurdle. Recent advances aimed at closing this gap include the development of more humanized disease models, and, while challenges persist, meaningful progress has been made. Advances include the identification of promising neuroprotective targets, greater insight into immune responses, and deeper characterization of neuron–glia–vascular interactions. Persistent challenges now center on mapping the disease microenvironment over time, optimizing cell replacement strategies, and identifying synergistic drug combinations.

Looking ahead, Catalyst for a Cure Team 3 will validate and compare prioritized neuroprotective targets across models and functional assays, exploring both neuron-intrinsic and extrinsic strategies as well as potential combination therapies. Catalyst for a Cure Team 4 will evaluate similarities and differences across diseases, models, and stages of degeneration to advance the feasibility of pan-disease neuroprotective strategies. Ultimately, these efforts aim to contribute both disease-specific and pan-disease therapies for glaucoma and other neurodegenerative diseases—transforming the treatment landscape in a manner similar to oncology. Just as cancer care has evolved through the development of many new therapies and combination approaches, the shared goal of CFC3 and CFC4 is to build a similarly robust arsenal of treatments that alleviate the progression and severity of neurodegenerative diseases.

## Data Availability

No datasets were generated or analysed during the current study.

## Abbreviations

AD: Alzheimer’s Disease
ALS: Amyotrophic lateral sclerosis
BAMs: [Central nervous system] border-associated macrophages
BBB: Blood-brain barrier
CAMs: CNS-associated macrophages
CFC3: Catalyst for a Cure Team 3
CFC4: Catalyst for a Cure Team 4
CRISPR: Clustered Regularly Interspaced Short Palindromic Repeats
CNS: Central nervous system
DAMs: Disease-associated microglia
FACS: Fluorescence-Activated Cell Sorting
FANS: Fluorescence-Activated Nuclear Sorting
FIND-Seq: Fast Identification of Nuclei using DNA barcodes Sequencing
HD: Huntington’s Disease
iPSC: Induced pluripotent stem cell
KO: Knockout
RGC: Retinal ganglion cell
PD: Parkinson’s Disease
MAP-Seq: Multiplexed Analysis of Projections by Sequencing
MS: Multiple sclerosis
NK: Natural killer cells
NSC: Neural stem cell
NPS: Neural progenitor cell
ONC: Optic nerve crush
OCT: Optical coherence tomography
RNA: Ribonucleic acid
RNAseq: RNA sequencing
RABID-Seq: Rabies Barcode Interaction Detection followed by Sequencing
scRNAseq: Single-cell RNA Sequencing
snRNAseq: Single-nucleus RNA Sequencing
SOHU: Silicone oil-induced ocular hypertension underdetected
SPEAC-Seq: Systematic Perturbation of Encapsulated Associated Cells followed by Sequencing
